# Scientific legacy of COVID-19 at the FMUSP-HC academic health system: current status and implications for the future

**DOI:** 10.6061/clinics/2021/e3630

**Published:** 2021-11-24

**Authors:** Geraldo Filho Busatto, Clovis Artur Silva, Antonio José Rodrigues Pereira, Eloisa Bonfá, Tarcísio Eloy Pessoa de Barros-Filho

**Affiliations:** Hospital das Clinicas HCFMUSP, Faculdade de Medicina, Universidade de Sao Paulo, Sao Paulo, BR.

Coronavirus disease 2019 (COVID-19) is a viral infection, with potentially devastating consequences to the health and welfare of human populations worldwide. It has had an enormous impact on the daily activities of the largest academic health system in Latin America, namely the Faculdade de Medicina da Universidade de São Paulo & Hospital das Clínicas (FMUSP-HC) system. From the beginning of the pandemic, research groups from the FMUSP-HC system conducted a wide range of medical investigations. These groups produced novel findings that have added to the global scientific database. This research will assist in mitigating the impacts of this new disease. Data compiled by the Observatório de Produção Intelectual (OPI) of the FMUSP-HC system (https://observatorio.fm.usp.br/) (1) indicate that there was an increase of over 10% in the total number of publications per year (n=3210) in 2020 compared with 2018 and 2019. A large number of these articles were focused on COVID-19 (n=334). In 2021, a total of 338 COVID-19 related articles were published until October 2021. This accounts for a large proportion (13.7%) of our annual scientific productivity. More than 50% of the COVID-related papers produced by members of the FMUSP-HC community included original data. A notable number of those articles have been published in the highest-impact international periodicals. These include Science, Nature Medicine, Journal of the American Medical Association, British Medical Journal, and the Lancet family of journals. Finally, the overall citability of our scientific COVID-19 publications can be attested to by an H-index of 35 up until October 2021.

The scientific output of the FMUSP-HC system regarding COVID-19 has also been enhanced by a number of novel institutional initiatives. These include: 1) the organization of electronic medical records that comprehensively document, for the purpose of research studies, hospital information on more than 5,000 COVID-19 cases treated at the Hospital das Clínicas (data on vaccinations as well as other health care initiatives offered to the thousands of workers based at the FMUSP-HC system are also documented); 2) large-scale analyses of laboratory tests and imaging results from patients with COVID-19; 3) initiation of biobank facilities to store large quantities of blood samples and *post-mortem* tissue from patients with COVID-19; 4) various innovative methodological strategies, including artificial intelligence, for interpretating computed tomography data; and 5) the development of cooperative, multidisciplinary programs to follow patients who were hospitalized and survived moderate to severe COVID-19. The health system also initiated a Steering Committee that took responsibility for the strategic oversight and governance of these institutional activities. This initiative brought together directors from all administrative research committees at the FMUSP-HC system. It also included representatives from the central clinical board and scientific committees of all institutes of the Hospital das Clínicas. Finally, COVID-related research initiatives led teams at the FMUSP-HC system that were financially supported by donations from the general public and private local companies. This was accomplished because of the innovative HC-COMVIDA organization (https://viralcure.org/c/hc) (2). This organization provided a timely, vital addition to the funds raised from local research support agencies.

The articles listed in the present issue of Clinics reflect the developments listed above. They address a wide range of scientific topics concerning COVID-19 and bring together authors from multiple departments of the Faculdade de Medicina da Universidade de São Paulo and institutes from the Hospital das Clínicas.

Most of the included papers were based on data obtained from patients treated for COVID-19 at the Hospital das Clínicas. One study showed that the mortality of patients with acute COVID-19 during in-hospital stays was associated with high D-dimer levels. These levels were more directly related to systemic inflammation than to the occurrence of venous thromboembolism (3). Another report identified that a greater extent of pulmonary involvement (lung lesion burden greater than 50% as assessed by computed tomography) was associated with a higher risk of mortality because of COVID-19 (4). An additional study reported observational findings from a sample of pediatric patients with laboratory-confirmed COVID-19. This investigation documented clinical characteristics of the acute disease and its’ risk factors for mortality in neonates, children, and adolescents (5). A further investigation reported a high frequency of new cardiovascular manifestations or decompensation of underlying heart conditions in adult patients hospitalized at the Hospital das Clínicas because of acute COVID-19. These patients were referred for specialized cardiac evaluation (6). Finally, an interventional trial demonstrated that a single dose of 200,000 IU of vitamin D3 did not significantly reduce the length of hospital stay in COVID-19 patients with severe 25-hydroxyvitamin D-deficiency (7).

Adding to the above studies, two reports evaluated the use of novel minimally invasive autopsy techniques applied to deceased patients with COVID-19. These techniques illustrated the feasibility of an extended ultrasound-guided strategy to increase the accuracy in detecting cardiopulmonary events involving the large vessels (8). It also documented the usefulness of *post-mortem* chest computed tomography imaging in fatal cases of pediatric and adult COVID-19 (9).

Regarding the follow-up of patients who survived hospital admission because of COVID-19, one investigation of adult patients showed a greater loss of physical and pulmonary capacity between two and four months post-discharge in patients with longer durations of hospitalization, intensive care unit stays, and intubations (10). Moreover, a follow-up investigation of pediatric patients with COVID-19 showed a high frequency of persistent COVID-related symptoms and a lower health-related quality of life in the physical and school domains of these patients (11).

Three additional articles exemplify the variety of impacts imposed by the COVID-19 pandemic on the overall functioning of our health system and wellbeing of the population. In the first of these papers, the gastric cancer surgery group at the *Hospital das Clínicas* reported and discussed changes to the number of surgeries and diagnostic procedures during the pandemic (12). The second manuscript discussed the potential risk for patients with COVID-19 to develop strongyloidiasis, a highly neglected parasitic disease (13). Finally, one study based on online survey methods showed that increased levels of screen time and intrafamilial violence during the first wave of the COVID-19 pandemic were associated with poor sleep quality and low health-related quality of life in both healthy and chronically immunosuppressed quarantined adolescents (14).

The COVID-19 pandemic also produced opportunities for the rapid implementation and testing of novel health-promoting strategies. This is another field of research addressed in the present issue of Clinics. Two articles evaluate the implementation of a program to protect the mental health of healthcare workers in the FMUSP-HC system. The first article discusses the feasibility of this program and provides evidence that preventive actions for healthcare staff should be focused on anxiety and depression, particularly in nursing staff (15). The second article discusses distinctions in the patterns of help-seeking and symptoms in physicians between the first and second waves of the pandemic (16).

Finally, vaccination (the most essential strategy to control COVID-19) is currently the subject of several large-scale research projects in the FMUSP-HC system. An editorial included in the present fascicle, produced by the Obstetrics team, provides compelling arguments to support the use of immunization to reduce both fetal and maternal morbidity related to COVID-19. This is especially true in Brazil, where the maternal death rates because of COVID-19 have been extremely high (17).

We foresee that the urgency to produce scientific information on COVID-19 will continue for a considerable amount of time. This is essential to meet the pressing demands of prevention, treatment, and rehabilitation for this new disease.

Moreover, in a broader sense, global data that are being amassed regarding the multiple impacts of COVID-19 should assist scientific communities worldwide in refining current models of why humans become ill and then recover. This scenario should also assist in consolidating new ways of conducting research. Higher levels of peer cooperation and greater support and participation from society will hopefully be present in the future. We hope that the content of this issue of Clinics will provide readers with an enlightening glimpse of FMUSP-HC’s auspicious scientific legacy and implications for the future.

## AUTHOR CONTRIBUTIONS

All authors contributed substantially to the conception and design of the study and in the analysis and interpretation of data. All authors critically revised the manuscript and approved its final version.
